# Automatic segmentation and volumetry of multiple sclerosis brain lesions from MR images

**DOI:** 10.1016/j.nicl.2015.05.003

**Published:** 2015-05-16

**Authors:** Saurabh Jain, Diana M. Sima, Annemie Ribbens, Melissa Cambron, Anke Maertens, Wim Van Hecke, Johan De Mey, Frederik Barkhof, Martijn D. Steenwijk, Marita Daams, Frederik Maes, Sabine Van Huffel, Hugo Vrenken, Dirk Smeets

**Affiliations:** a icometrix, R&D, Leuven, Belgium; b Department of Neurology, Center for Neurosciences, Universitair Ziekenhuis Brussel, Vrije Universiteit Brussel (VUB), Brussel, Belgium; c Department of Radiology and Nuclear Medicine, Neuroscience Campus Amsterdam, VU University Medical Center, Amsterdam, The Netherlands; d Department of Electrical Engineering—ESAT, PSI Medical Image Computing, KU Leuven, Leuven, Belgium; e Department of Electrical Engineering—ESAT, STADIUS Center for Dynamical Systems, Signal Processing and Data Analytics, KU Leuven, Leuven, Belgium; f Medical IT, iMinds, Leuven, Belgium; g Department of Anatomy and Neurosciences, Neuroscience Campus Amsterdam, VU University Medical Center, Amsterdam, The Netherlands; h Department of Physics and Medical Technology, Neuroscience Campus Amsterdam, VU University Medical Center, Amsterdam, The Netherlands

**Keywords:** Multiple sclerosis, Magnetic resonance imaging, White matter lesions, Brain segmentation

## Abstract

The location and extent of white matter lesions on magnetic resonance imaging (MRI) are important criteria for diagnosis, follow-up and prognosis of multiple sclerosis (MS). Clinical trials have shown that quantitative values, such as lesion volumes, are meaningful in MS prognosis. Manual lesion delineation for the segmentation of lesions is, however, time-consuming and suffers from observer variability. In this paper, we propose MSmetrix, an accurate and reliable automatic method for lesion segmentation based on MRI, independent of scanner or acquisition protocol and without requiring any training data. In MSmetrix, 3D T1-weighted and FLAIR MR images are used in a probabilistic model to detect white matter (WM) lesions as an outlier to normal brain while segmenting the brain tissue into grey matter, WM and cerebrospinal fluid. The actual lesion segmentation is performed based on prior knowledge about the location (within WM) and the appearance (hyperintense on FLAIR) of lesions. The accuracy of MSmetrix is evaluated by comparing its output with expert reference segmentations of 20 MRI datasets of MS patients. Spatial overlap (Dice) between the MSmetrix and the expert lesion segmentation is 0.67 ± 0.11. The intraclass correlation coefficient (ICC) equals 0.8 indicating a good volumetric agreement between the MSmetrix and expert labelling. The reproducibility of MSmetrix' lesion volumes is evaluated based on 10 MS patients, scanned twice with a short interval on three different scanners. The agreement between the first and the second scan on each scanner is evaluated through the spatial overlap and absolute lesion volume difference between them. The spatial overlap was 0.69 ± 0.14 and absolute total lesion volume difference between the two scans was 0.54 ± 0.58 ml. Finally, the accuracy and reproducibility of MSmetrix compare favourably with other publicly available MS lesion segmentation algorithms, applied on the same data using default parameter settings.

## Highlights

• MSmetrix is a new automatic method for white matter lesion segmentation based on MRI.
• MSmetrix performs unsupervised segmentation using 3D T1-weighted and FLAIR MR images*.*
• MSmetrix is validated on two MS datasets and shows good accuracy and reproducibility.
• MSmetrix compares favourably with other publicly available MS lesion segmentation algorithms.

## 1 Introduction

Multiple sclerosis (MS) is the most common inflammatory demyelinating disease of the central nervous system that is characterised by the presence of white matter (WM) lesions (Compston and Coles, 2008). These WM lesions are visible on a magnetic resonance imaging (MRI) brain scan and appear hyper-intense on T2-weighted or Fluid Attenuated Inversion Recovery (FLAIR) images and some appear hypo-intense on T1-weighted images. Brain MRI scans are part of the clinical routine of MS patients for both diagnosis and follow-up (Polman et al., 2011). In clinical practice, typically, lesions are only visually assessed. However, clinical trials have shown that lesion volumes are meaningful outcomes for disease prognosis (Tur et al., 2011). Accurately measuring lesion volumes is, therefore, of considerable interest in clinical practice. Manual segmentation of MS lesions is time consuming and suffers from large intra- and inter-observer variability. Therefore, in clinical trials and research studies, semi-automated methods are increasingly used. The ultimate aim, however, is to use a fully automated lesion segmentation approach, as it can further decrease the observer dependency as well as the time needed from the expert. This will be especially important for large clinical trials, as a huge amount of data needs processing. For clinical practice, an automated method would make it possible to measure lesion volumes, thereby further standardising and quantifying the MRI reading.

In this paper, MSmetrix is introduced, a robust automatic method for WM lesion segmentation based on 3D T1-weighted and 3D FLAIR MR images. The method is independent of scanner and acquisition protocol and does not require a training image database of expert lesion segmentations. The proposed method models the WM lesions as an outlier class while classifying the brain into three classes, grey matter (GM), WM and cerebrospinal fluid (CSF) using a healthy brain atlas.

The aim of this paper is to validate the proposed lesion segmentation method on two distinct datasets from two clinical centres, used to assess accuracy as well as reproducibility. In addition, results are compared both quantitatively and qualitatively with two well-known and publicly available automatic unsupervised lesion segmentation software implementations: LST (Schmidt et al., 2012) and Lesion-TOADS (Shiee et al., 2010).

## 2 Methods

This section provides more details on the proposed lesion segmentation method and summarises the publicly available lesion segmentation methods we compare to. Furthermore, the data and the validation tests used for evaluation of the methods are described.

### 2.1 Method description

**Fig. 1:**
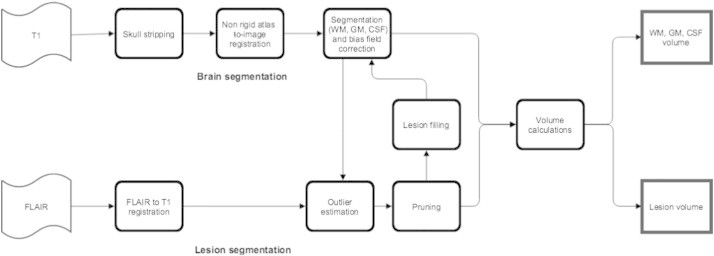
Schematic representation of the MSmetrix method.

Fig. 1 shows a schematic overview of the proposed method, which takes as inputs 3D T1-weighted and a 3D FLAIR image acquired from an MS patient. The images are preprocessed and co-registered before executing the main loop of the algorithm, consisting of brain tissue segmentation, lesion segmentation and lesion filling. We describe each of the steps into more detail below.

The first step, the preprocessing, has three stages:

1 In the first stage, the input FLAIR image of the patient is rigidly co-registered with the input T1-weighted image using the normalised cross correlation coefficient as a similarity measure.
2 In the second stage, the T1-weighted input image is skull stripped classifying each voxel either as a brain region or a non-brain region based on the affine registration of a brain mask available from the MNI-atlas using the normalised cross correlation coefficient as a similarity measure followed by a non-rigid registration using the normalised mutual information as a similarity measure.
3 In the third stage, the probabilistic anatomical priors for GM, WM and CSF, which are also available from the MNI-atlas, are transferred to the skull stripped T1-weighted image space using the affine transformation and non-rigid deformation computed above.

In these preprocessing stages, the rigid and affine registrations use a multi-resolution approach with a Trimmed Least Square scheme and block-matching (NiftyReg, Ourselin et al., 2002). The non-rigid registration is based on the Free-Form B-spline deformation model and also uses a multi-resolution approach (NiftyReg, Modat et al., 2010).

In the second step, which builds further on the work of Van Leemput et al. (1999), a probabilistic model is formulated to segment the skull stripped T1-weighted image based on prior knowledge given by the probabilistic tissue priors mentioned above. The model assumes a Gaussian distribution of the image intensities per tissue class, a smoothly varying bias field for the intensity non-uniformities and contains a spatial consistency model based on a Markov Random Field (MRF). This model is optimised using an expectation maximisation (EM) algorithm (Van Leemput et al., 1999) as implemented in NiftySeg (Cardoso, 2012). The algorithm iteratively estimates the parameters of each tissue class, as well as the bias field parameters, and maintains the spatial consistency until convergence. After the convergence of the EM algorithm, the T1-weighted image is bias field corrected and segmented into the three tissue classes, i.e., GM, WM and CSF.

In the third step, an outlier class is estimated from the co-registered FLAIR image of the same patient using the three tissue class segmentations from the previous step as prior information. This is performed using the same EM algorithm as described in the second step, but now an outlier map is included (Van Leemput et al., 2001). Using the tissue segmentations of the T1-weighted image as prior information, the intensities of each tissue class in the FLAIR image are modelled as a normal distribution and the deviation of each FLAIR voxel from this model is estimated as an outlier belief map. This map is iteratively updated by the EM algorithm and, after convergence, the FLAIR image is bias field corrected and an outlier belief image is produced. This outlier belief image is used as an initialisation for the MS lesion segmentation in the next step.

In the fourth step, we segment the lesions in the outlier map, i.e., we ‘prune’ the outlier map, as not every outlier is a lesion (e.g., the outlier map might include partial volume effects, artefacts, etc.). In order to differentiate the MS lesions from such non-lesion outliers, some extra a priori information about the location and the appearance of the lesions needs to be incorporated. The outliers need to be in the WM region, where the WM segmentation is derived from T1-weighted image segmentation in the second step. Moreover, the underlying intensities of the outliers should be hyperintense compared to the GM intensities on bias field corrected FLAIR image. The hyperintensity is defined as a threshold that is equal to the mean plus two times the standard deviation of GM intensities in the bias field corrected FLAIR image. Furthermore, another mask based on co-registered T1-weighted image atlas, is used to exclude areas (WM in-between the ventricles and in the cortical regions near the great longitudinal fissure) that are prone to show false lesion segmentation. Finally, each masked outlier voxel needs to have a minimum number of 5 adjacent outlier voxels (empirically determined), in order to avoid spurious lesion detection.

In the fifth step, this lesion segmentation is then used to fill in the lesions in the bias corrected T1-weighted image with WM intensities. This global lesion filling method is very similar to the LEAP method (Chard et al., 2010) where the mean and standard deviation of WM intensities are computed using the WM segmentation from the second step. In our case, the WM segmentation is a soft segmentation and therefore, is thresholded to values above 0.5, in order to make sure that only WM intensities are included in the statistics. Finally, the whole filled-in volume is smoothed with a Gaussian kernel of radius 1 voxel, and normalisation is applied in order to restore the global standard deviation of the WM intensities as in Chard et al. (2010).

Steps 2–5 are repeated until there is no significant change in the tissue and the lesion segmentation. The idea of repeating the second and the third step is that the lesions are primarily WM, therefore, the T1-weighted lesion filling will result in better brain tissues segmentation, which in turn results in better segmentation of lesions due to the improved WM mask used in the fourth step. After the last iteration lesions are recovered from the GM segmentation in case the outlier belief is high. This is because some WM lesions that are very close to cortical GM might be wrongly segmented as GM and therefore, are missed in the pruning step, i.e. the fourth step. These lesions are added to the previously found lesions in the fourth step. Subsequently the T1-weighted image is filled one more time and segmented again providing the final segmentations of WM, GM and CSF.

### 2.2 Other methods

For comparison, the validation tests performed for MSmetrix were executed on the same datasets using two state-of-the-art software packages: LST (Schmidt et al., 2012) and Lesion-TOADS (Shiee et al., 2010). Unless otherwise stated, default parameter settings were used for all implementations, thus parameter tuning was not performed neither at dataset level, nor at patient level.

#### 2.2.1 LST

LST (Lesion Segmentation Tool) v1.2.3 is implemented in SPM8 (http://www.fil.ion.ucl.ac.uk/spm/software/spm8/) and is based on a lesion growth algorithm described in Schmidt et al. (2012). In short, LST determines GM, WM and CSF segmentations from T1-weighted images and computes the FLAIR intensity distributions of these tissue classes. The amount of ‘hyperintensity’ of each voxel in terms of distance from the mean intensity of the WM, GM and CSF distributions in the FLAIR image is crucial for defining a conservative lesion belief map (obtained by thresholding the GM belief map) and a liberal lesion belief map (consisting of the sum of the three lesion belief maps). Lesion growing is then performed iteratively between the conservative and the liberal belief maps, until no more voxels are added to the lesions.

#### 2.2.2 Lesion-TOADS

Lesion-TOADS (Shiee et al., 2010) is available as a plug-in for the MIPAV software (http://mipav.cit.nih.gov/). Lesion-TOADS implements an iterative algorithm for fuzzy classification of the image intensities, using a combination of topological and statistical atlases. An additional lesion class is added to the brain segmentation model, using the same spatial prior as WM; lesions and WM are then separated by selecting, inside the grouped region, whichever has the higher membership value. Prior knowledge about areas where false positives commonly occur is used to define penalty weights based on the distance to these areas (e.g., distance to ventricles, GM structures and interventricular WM). This method segments multichannel input images simultaneously, using an intensity-weighing scheme that optimises the effect of each channel onto the segmentation of each tissue class. Although it is not a default setting, the method is also able to estimate bias field corrections to deal with local intensity non-uniformities. For a fair comparison against MSmetrix and LST, where bias field corrections are included by default, this option was also turned on for Lesion-TOADS, since otherwise its results were not competitive.

### 2.3 Data

#### 2.3.1 Dataset 1

20 MS patients participated in a study at VU University Medical Center, Amsterdam, the Netherlands. The study was approved by the local ethics committee and all patients signed informed consent forms. MR imaging was performed on a 3 T whole body scanner (GE Signa HDxt, Milwaukee, WI, USA). The protocol contained two 3D sequences: a fat-saturated 3D FLAIR (TR: 8000 ms, TE: 125 ms, TI: 2350 ms, 250 × 250 mm^2^ field of view (FOV), 132 sagittal slices, 0.98 × 0.98 × 1.2 mm^3^ voxel resolution) and a 3D T1-weighted fast spoiled gradient echo (FSPGR) sequence (TR 7.8 ms, TE 3 ms, FA 12°, 240 × 240 mm^2^ FOV, 176 sagittal slices, 0.94 × 0.94 × 1 mm^3^ voxel resolution). Reference WM lesion segmentations were constructed manually using the 3D FLAIR and 3D T1-weighted images by a highly trained neuroradiological team (Steenwijk et al., 2013). In short, both 3D T1-weighted and 3D FLAIR images were co-registered and orthogonally reformatted to the axial plane, and the axially reformatted images were then used to identify and outline the lesions. Lesion identification was performed by three raters in consensus using the 3D FLAIR images; the raters were allowed to view the corresponding co-registered 3D T1-weighted image. Lesions were only identified if they were larger than 3 voxels in-plane and visible on at least two consecutive slices. In the next step, two trained technicians manually outlined the identified lesions on the 3D FLAIR using MIPAV (http://mipav.cit.nih.gov). The expert lesion segmentation resulted in a wide range of lesion volumes going from 1.88 to 50.95 ml, with a mean of 16.33 ml and a standard deviation of 11.49 ml. For comparison purpose, these lesion segmentations were resampled from the FLAIR image space to their corresponding T1-weighted image space using nearest neighbour interpolation as implemented in NiftyReg (Modat, 2010).

#### 2.3.2 Dataset 2

10 MS patients participated in a study at UZ Brussels, Brussels, Belgium. The study was approved by the local ethics committee and all patients signed informed consent forms. MR imaging was performed for each patient twice on 3 T whole body scanners from three different manufacturers (GE Medical Systems Discovery MR750w; SIEMENS Skyra; Philips Medical Systems Achieva). The patient went out of the scanner after the first scan and was re-positioned for the second scan. The GE scanner protocol contained, among others, two 3D sequences: a fat-saturated 3D FLAIR (TR: 9500 ms, TE: 135.78 ms, TI: 2428.0 ms, 240 × 240 mm^2^ field of view (FOV), 232 sagittal slices, 0.4688 × 0.4688 × 0.7 mm^3^ voxel resolution) and a 3D T1-weighted FSPGR sequence (TR 7.32 ms, TE 3.144 ms, FA 12°, 220 × 220 mm^2^ FOV, 328 sagittal slices, 0.4297 × 0.4297 × 0.5 mm^3^ voxel resolution). The SIEMENS scanner protocol contained, among others, two 3D sequences: a fat-saturated 3D FLAIR (TR: 5000 ms, TE: 387.0 ms, TI: 1800.0 ms, 230 × 230 mm^2^ field of view (FOV), 192 sagittal slices, 0.4492 × 0.4492 × 0.9 mm^3^ voxel resolution) and a 3D T1-weighted MPRAGE sequence (TR 2300 ms, TE 2.29 ms, FA 8°, 240 × 240 mm^2^ FOV, 176 sagittal slices, 0.9375 × 0.9375 × 0.94 mm^3^ voxel resolution). The PHILIPS scanner protocol contained, among others, two 3D sequences: a fat-saturated 3D FLAIR (TR: 4800 ms, 240 × 240 mm^2^ field of view (FOV), 321 sagittal slices, 1.0416 × 1.0416 × 0.56 mm^3^ voxel resolution) and a 3D T1-weighted FSPGR sequence (TR 4.936 ms, FA 8°, 230 × 230 mm^2^ FOV, 310 sagittal slices, 0.5324 × 0.5324 × 0.5 mm^3^ voxel resolution). For this dataset, no expert segmentations were available. Also, the resolution of T1-weighted and FLAIR images from all the scanners is high and therefore, due to very high computational memory requirement, none of the methods were able to run on these high resolution images; therefore, the T1-weighted image was down sampled to (1 × 1 × 1 mm^3^) resolution. The FLAIR image was not down sampled at this point because it is rigidly co-registered to T1-weighted image in the initial stage of the method and thus will have the T1-weighted image resolution.

### 2.4 Performance tests

#### 2.4.1 Comparison to expert segmentations on dataset 1

For dataset 1, the agreement between automatic methods and expert reference segmentation is evaluated at a voxel-by-voxel level. Spatial agreement is reported by the Dice similarity index (Dice, 1945), defined as the ratio between the number of voxels where both the automatic and the expert reference segmentation agree (true positives) and the mean number of voxels labelled as lesion by the two methods. Additionally for dataset 1, the average Dice performance for each method is computed separately for patients with small, medium and large lesion volumes in order to assess whether the methods' performance is depending on lesion volume. For the total lesion volume, agreement between automatic and expert reference segmentation is evaluated through the intra-class correlation coefficient (ICC) and the absolute volume difference. ICC is a measure assessing the agreement of measurements made by multiple observers measuring the same quantity (Koch, 1982). In this paper, ICC is used in the absolute agreement formulation. The absolute volume difference is computed as the absolute difference between the total volume reported by the automatic method and the corresponding value derived from expert reference segmentation.

For dataset 1, the automatic methods segmentation quality is evaluated at a voxel-by-voxel level. The ability to segment lesions is reported by sensitivity and is defined as the ratio between true positives and the total number of lesion voxels in the expert reference segmentation (true positives and false negatives). The relevance of the segmentation is measured by precision and is defined as the ratio between true positives and the total number of lesion voxels in the automatic segmentation (true positives and false positives). Since MSmetrix is an iterative method, the benefit gained over the iterations is verified by reporting the Dice similarity index for dataset 1. To determine if there is a statistical difference between MSmetrix and LST and between MSmetrix and Lesion-TOADS methods' performance, two tailed paired t-test is performed, except for the average Dice performance as a function of lesion volume, where the sample size is small.

#### 2.4.2 Lesion volume reproducibility assessment on dataset 2

For dataset 2, ten patients were examined twice in each scanner. For the total lesion volume, agreement between the first and the second scan for each scanner is evaluated through Dice similarity index and absolute volume difference as described in Section 2.4.1. Here, two tailed paired Wilcoxon signed-rank test is performed instead of t-test as the Dice similarity index and absolute volume difference for all methods are not normally distributed.

## 3 Results

### 3.1 Accuracy validation (dataset 1)

In this section we first present quantitative results (Dice similarity index, ICC and absolute volume difference) followed by the qualitative results, where the segmentation results are evaluated visually by presenting the best and the worst cases for each of the automatic methods.

#### 3.1.1 Quantitative results

Table 1 presents the quantitative results where the Dice similarity index reaches an average of 0.67 for MSmetrix, followed by 0.61 for Lesion-TOADS and 0.55 for LST.

**Fig. 2:**
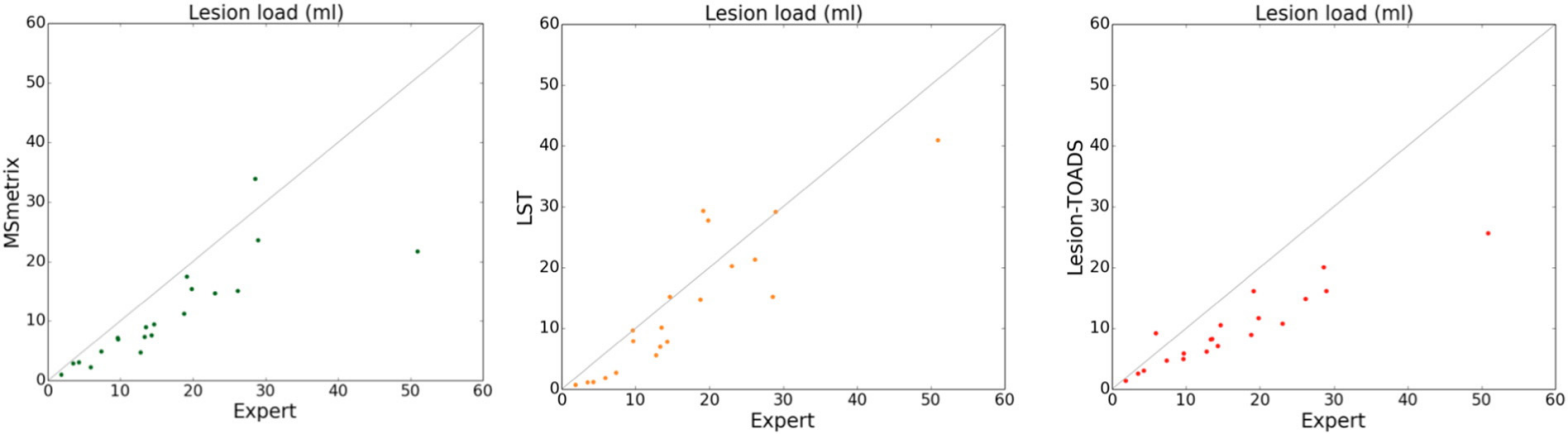
Scatter plots of expert reference values versus automatically computed values for total lesion volume (ml). The three columns show results for MSmetrix, LST and Lesion-TOADS, respectively.

In order to visualise the volumetric correlation of each automatic method to the expert reference segmentation, Fig. 2 shows the scatter plots for total lesion volume (lesion load) in ml of each method compared to the volume of expert reference segmentation. MSmetrix is well correlated to the expert reference lesion volume, but has a general trend of slightly underestimating the lesion volume compared to the expert reference lesion segmentation. Lesion-TOADS shows an even stronger trend of volume underestimation. The absolute volume error and total lesion volume are better correlated to the expert reference values for LST (average 4.75 ml absolute volume error with an ICC of 0.87) than for MSmetrix and Lesion-TOADS (5.15 ml, 0.80, and 6.8 ml, 0.63 respectively).

In order to provide a better understanding of the segmentation performance, also the sensitivity and precision at voxel level are presented in Table 2 for each of the automatic methods compared to the expert reference segmentation. The sensitivity reaches an average of 0.57 for MSmetrix, followed by 0.50 for Lesion-TOADS and for LST. MSmetrix is more precise, with an average precision of 0.83 compared to 0.70 and 0.81 for LST and Lesion-TOADS, respectively.

Table 3 presents the average Dice performance of each method for patients with small, medium and large lesion volumes, computed according to the expert reference segmentation. For this, the dataset was divided into three intervals according to lesion load: <5 ml, 5–15 ml and >15 ml. From the table it can be concluded that all methods show an increase in their performance for large lesion volume, with MSmetrix being most consistent, i.e. having a higher stable range of values among the groups.

The Dice similarity index for MSmetrix grows from 0.51 ± 0.14 at iteration 1, to 0.60 ± 0.13 at iteration 2, to 0.66 ± 0.11 at iteration 3, which proves the benefit of using the proposed iterative approach (see Fig. 1). Stopping after iteration 3 provides a reasonable trade-off between sensitivity and precision.

#### 3.1.2 Qualitative results

In the following figures of this section, the original FLAIR image is shown followed by the lesion segmentation from the expert reference segmentation (yellow), MSmetrix (green), LST (orange) and lesion-TOADS (red) are super-imposed on the bias corrected FLAIR images.

**Fig. 3:**
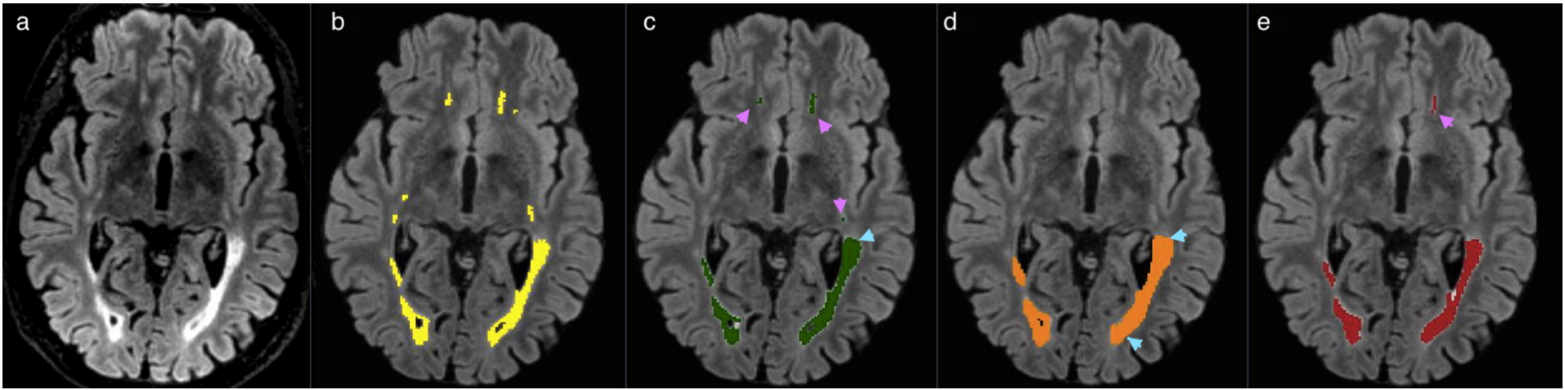
Original FLAIR image (a) followed by bias corrected FLAIR image and super-imposed lesion segmentation from: (b) expert segmentation, (c) MSmetrix, (d) LST, and (e) Lesion-TOADS. Cyan arrow heads show false positive lesions and overestimation of the lesion boundaries in LST. Pink arrow heads show lesions picked by MSmetrix but not by the other methods except one in Lesion-TOADS.

Fig. 3 shows the best case for MSmetrix (Dice: 0.84, sensitivity: 0.84 and precision: 0.83), which is also the best case for Lesion-TOADS (Dice: 0.79, sensitivity: 0.73 and precision: 0.87). This case has Dice: 0.69, sensitivity: 0.87, and precision: 0.57 for LST. The high sensitivity and low precision of LST are caused by the presence of false positive lesions and the overestimation of lesion boundaries (marked by cyan arrow heads) compared to the other two methods. Between MSmetrix and Lesion-TOADS, the higher Dice similarity index of MSmetrix is because of the higher sensitivity. Notice that the lesions marked by pink arrow heads were picked up by MSmetrix but not by the other methods except one by Lesion-TOADS.

**Fig. 4:**
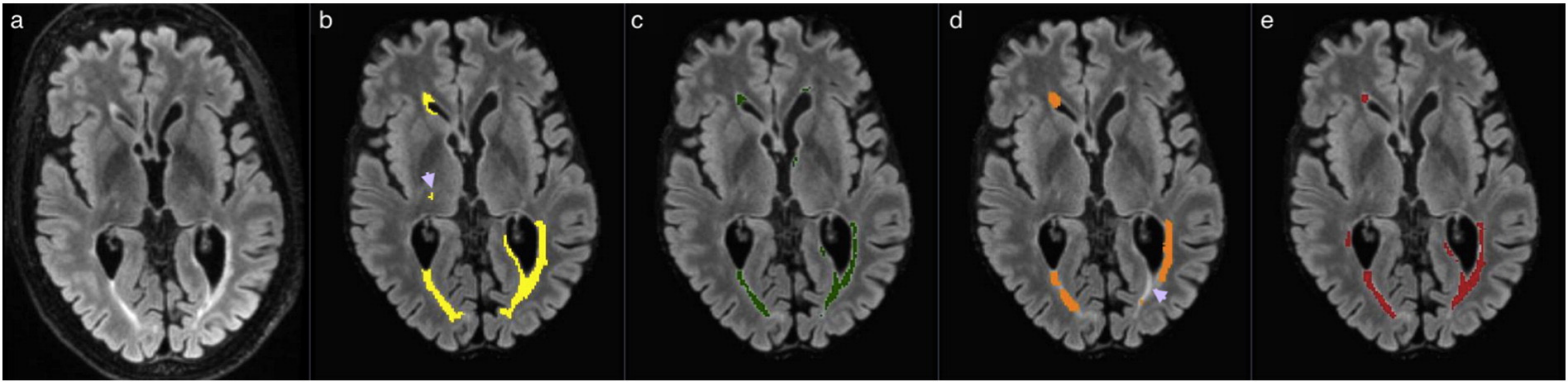
Original FLAIR image (a) followed by bias corrected FLAIR image and super-imposed lesion segmentation from: (b) expert segmentation, (c) MSmetrix, (d) LST, and (e) Lesion-TOADS. Purple arrow heads show some subtle lesions that are either missed or the lesions are underestimated.

Fig. 4 shows the worst case for MSmetrix (Dice: 0.45, sensitivity: 0.31 and precision: 0.79). LST and Lesion-TOADS had comparable performance, namely, Dice: 0.43, sensitivity: 0.31, and precision: 0.70 for LST, and Dice: 0.47, sensitivity: 0.35, and precision: 0.73 for Lesion-TOADS. Some subtle lesions are either missed or the lesions are underestimated (purple arrow head). This accounts for the low sensitivity and the low Dice similarity index for all the methods.

More examples from dataset 1 are given in the supplementary material.

### 3.2 Reproducibility assessment (dataset 2)

In this section, we first present quantitative results (Dice similarity index and absolute volume difference) followed by the qualitative results, where visual results are presented for MSmetrix, LST and Lesion-TOADS.

#### 3.2.1 Quantitative results

Table 4 presents the quantitative results where the Dice similarity index reaches an average of 0.71 for LST, followed by 0.69 for MSmetrix and 0.63 for Lesion-TOADS. The absolute lesion volume difference is less for LST (average 0.44 ml absolute volume difference) than for MSmetrix and Lesion-TOADS (0.54 and 1.58 ml respectively).

**Fig. 5:**
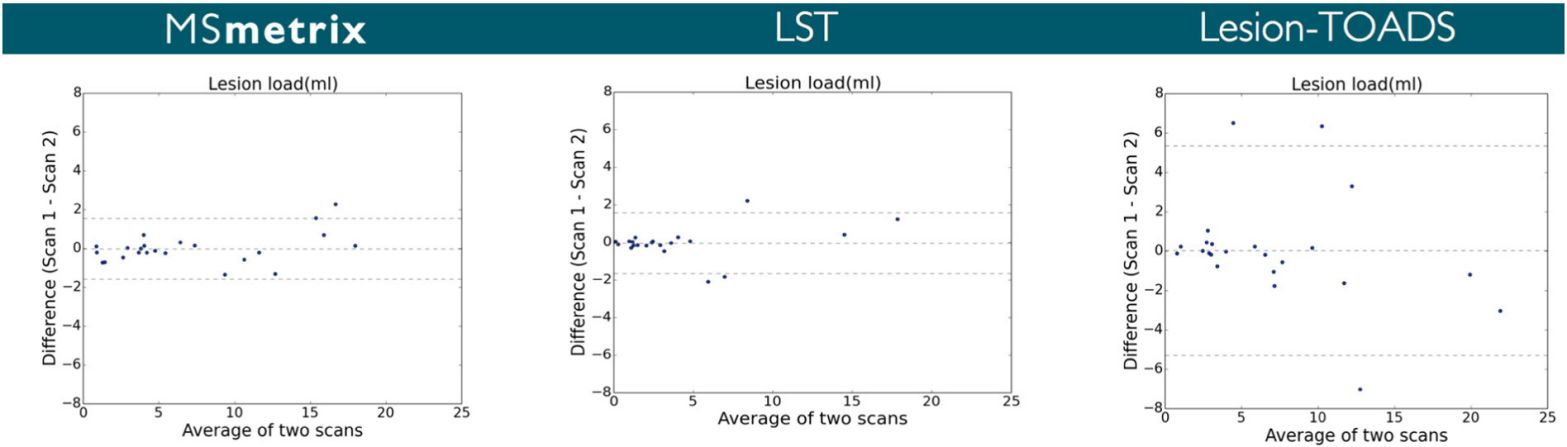
Bland–Altman plots for total lesion volume agreement between scan 1 and scan 2 of the corresponding automatic methods.

In order to visualise the volumetric agreement between scan 1 and scan 2 of the corresponding automatic methods, Fig. 5 contains the Bland–Altman plot for total lesion volume of each method at scan 1 compared to the volume at scan 2.

#### 3.2.2 Qualitative results

In the following figures of this section, the original FLAIR image is shown followed by the lesion segmentations from MSmetrix (green), LST (orange) and Lesion-TOADS (red) are super-imposed on the bias corrected FLAIR images. In each figure, the first row corresponds to the lesion segmentation of scan 1 and the second row corresponds to the lesion segmentation of scan 2.

**Fig. 6:**
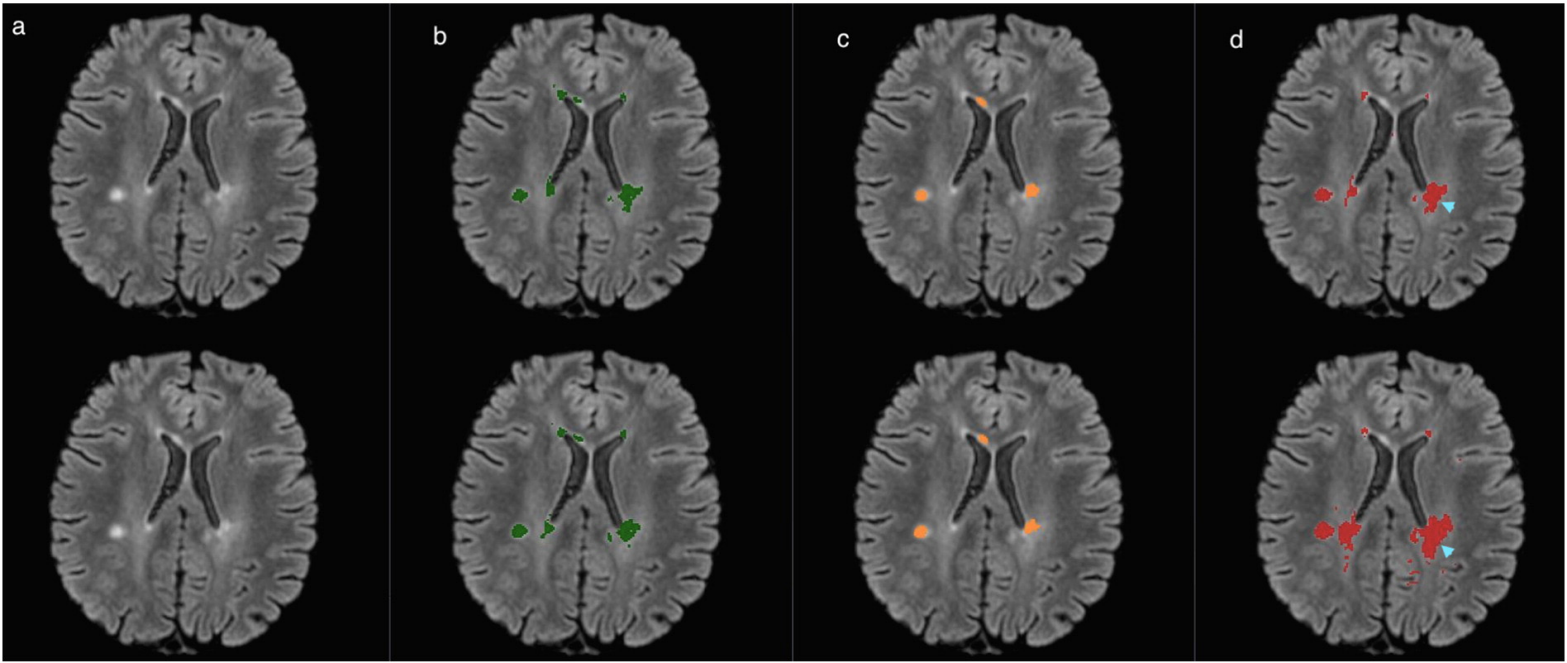
Bias corrected FLAIR image (a) and super-imposed lesion segmentation from: (b) MSmetrix, (c) LST, and (d) Lesion-TOADS. The first row corresponds to the lesion segmentation of scan 1 and the second row corresponds to the lesion segmentation of scan 2. Cyan arrow heads show the difference in the lesion segmentation boundary between scan 1 and scan 2 for Lesion-TOADS.

Fig. 6 shows the best case (highest reproducibility) for MSmetrix (Dice: 0.84). In this case, LST has the same performance (Dice: 0.84) as MSmetrix followed by Lesion-TOADS (Dice: 0.65). A good Dice similarity index for both MSmetrix and LST is mainly due to the fact that both methods are consistent in the lesion segmentation in their respective scan 1 and scan 2, while Lesion-TOADS seems to differ more in its lesion segmentation boundary between scan 1 and scan 2 (marked by cyan arrow head) and thus resulting in a lower Dice similarity index.

**Fig. 7:**
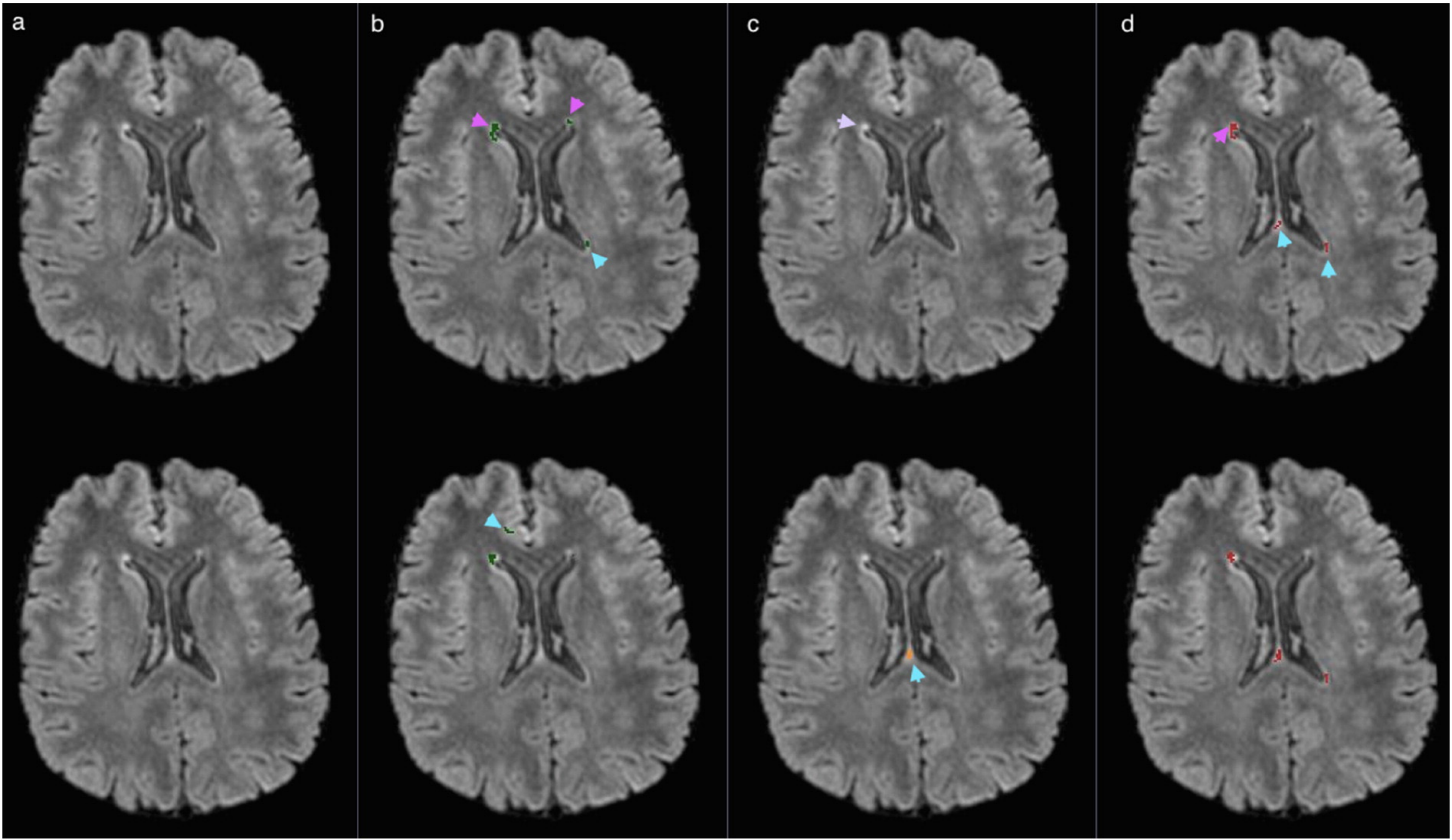
Bias corrected FLAIR image (a) and super-imposed lesion segmentation from: (b) MSmetrix, (c) LST, and (d) Lesion-TOADS. The first row corresponds to the lesion segmentation of scan 1 and the second row corresponds to the lesion segmentation of scan 2. Cyan arrow heads show the difference in the lesion segmentation between scan 1 and scan 2 for MSmetrix, LST and Lesion-TOADS. Pink arrow heads show subtle lesions that are picked up by MSmetrix and Lesion-TOADS. Purple arrow head shows missed subtle lesions by LST.

Fig. 7 shows the worst case for MSmetrix (Dice: 0.38). In this case, Lesion-TOADS has the best performance (Dice: 0.52) followed by LST (Dice: 0.46). The low Dice similarity index for MSmetrix is caused by false lesion detection in either of the scans (marked by cyan arrow heads). The higher Dice similarity index for Lesion-TOADS compared to MSmetrix and LST is mainly due to its quite consistent performance in detecting both true and false lesions in scan 1 and scan 2 (marked by pink and cyan arrow heads) for this case. For LST, a lower Dice similarity index is mainly due to the fact that it detects several few false lesions in either of the scans (marked by cyan arrow heads). However, both MSmetrix and Lesion-TOADS are more sensitive and thus detect subtle lesions (marked by pink arrow heads) whereas LST misses them in most of the cases (marked by purple arrow head).

More examples from dataset 2 are given in the supplementary material.

## 4 Discussion and conclusion

in this paper, a robust method for WM lesion segmentation is proposed, incorporating a lesion-filling algorithm for T1-weighted images and an iterative process of using the lesion-filled T1-weighted image for WM/GM/CSF tissue segmentation and the FLAIR image for lesion detection and segmentation. The method is fully automatic and has proven to be robust for different scanners without parameter tuning. As opposed to previous work (Van Leemput et al., 2001; Shiee et al., 2010), where multi-channel images were used simultaneously for lesion segmentation, we adopt an approach that tries to imitate more the human expert. In our case, T1-weighted and FLAIR images are used independently in order to fully exploit the main characteristics of each modality. In this paper, the method is evaluated for 3D T1-weighted and 3D FLAIR MR images. However, first visual assessment of the method's performance with 3D T1-weighted and 2D FLAIR MR images indicates good results.

The problem of MS lesion segmentation has received interest in the research community for the past 15 years (see García-Lorenzo et al., 2013; Mortazavi et al., 2012 for recent reviews). Although WM lesions are visible on the FLAIR image, automatic detection and delineation remain very challenging. The differentiation of MS lesions from ‘dirty’ WM and CSF pulsation artefacts in FLAIR is very difficult, as they share typical spatial locations and appear similar to MS lesions. Moreover, the MS lesions that are visible on FLAIR images exhibit very high pixel intensity variations, thus lesions can be classified as hyper- or hypo-intense. While it is quite easy to identify hyper-intense lesions, for example by intensity thresholding, hypo-intense lesions, on the other hand, are iso-intense with GM intensities and thus pose a challenge.

Manual lesion segmentation is time-consuming and suffers from inter- and intra-rater variability, with studies reporting low variability of total lesion volume estimation only in ideal situations, e.g., when lesion detection is done by a single observer and lesion delineation is performed by multiple raters, aided by the same semi-automated software tool (Filippi et al., 1998: coefficient of variation of 2.3% for intra-observer and 2.9% for inter-observer). However, such good results are not reproduced in the realistic situation when lesion detection and delineation are done by experts in different centres; as an example, MR images from 24 MS patients have been segmented by 2 experts for the MICCAI (2008) challenge (http://www.ia.unc.edu/MSseg/) and variability reached a relative volume difference of 68% and a percentage of false negatives (number of lesions marked by one rater only) of 32%, as reported by García-Lorenzo et al. (2013); section 5.3. Semi-automatic lesion segmentation could mean that lesion detection is done by the expert user and an automatic method for ‘lesion growing’ (i.e., lesion segmentation) is applied afterwards. This approach is more consistent than manual delineation, but still time consuming and not reproducible.

Automatic methods have the obvious advantage of being consistent and fast when compared to manual or semi-automatic methods (Vrenken et al., 2013). Some of the automatic lesion segmentation methods belong to the family of supervised classification methods, for which a representative training dataset, including expert segmentation, is required in order to build a model that can be used on new patients for lesion segmentation. Depending on the features extracted from images (local gradient intensity, mean intensity, spatial information, etc.) and on the type of classifier (k-nearest neighbours, artificial neural networks, Bayesian learning, support vector machines, etc.), many variants have been proposed. Although excellent results can be obtained with supervised classification on the training dataset, these methods have two disadvantages. The first difficulty lies in building a training dataset that encompasses MS lesions of all possible shapes, intensities and are heterogeneously distributed in the WM. The second non-trivial problem lies in pre-processing a new image (acquired on a different scanner than the one used for the training dataset), such that it matches the characteristics of the training dataset, e.g., by intensity normalisation. In other words, supervised methods perform well only when the new image to be segmented is well represented in the training dataset.

Another family of methods is based on unsupervised classification and does not require training images. Our proposed method, as well as LST and Lesion-TOADS, belong to this class. These methods are usually based on stochastic modelling of voxel intensity distribution. They perform brain segmentation into GM, WM and CSF (with or without lesion detection) and often rely on post-processing approaches in order to segment lesions (e.g., lesion growing or pruning). The assumptions that are made in order to segment lesions have a great impact on the results. From this point of view, MSmetrix has similarities to LST (Schmidt et al., 2012), since both methods detect FLAIR-hyper-intense outliers, which are further promoted as lesions according to their spatial probability of being in the WM, where the WM segmentation is basically derived from T1-weighted image segmentation. Nevertheless, MSmetrix' iterative process is meant to allow a more reliable estimation of the WM mask, as the WM/GM/CSF segmentation of the lesion-filled T1-weighted image should improve at each iteration. Lesion-TOADS, on the other hand, employs a sophisticated mechanism of combining information from different MR sequences (T1-weighted, T2, PD or FLAIR) in order to simultaneously segment lesions and brain structures, while distance maps from the boundaries of structures such as CSF are used to confine the segmented lesions to typical locations. These mechanisms are bound to have a large number of parameters that require tuning. The effect of keeping all parameters to their default values was obvious when comparing results of Lesion-TOADS on datasets 1 and 2. Dataset 2 images were not as good as those from dataset 1 (based on visual quality control of input images), which translated into a significant decrease in segmentation performance of Lesion-TOADS in dataset 2. Based on visual quality control of the resulting segmentations, Lesion-TOADS introduced a large amount of false positives, which was not the case in dataset 1.

An important contribution of this work is the experimental validation of MSmetrix, both for accuracy and reproducibility. On dataset 1, which was used for the evaluation of the segmentation accuracy, we could observe that MSmetrix has a reasonably good overlap with the expert segmentation, but with some systematic undersegmentation. The fair sensitivity of MSmetrix suggests that it can segment subtle lesions reasonably well but still misses small lesions (lesions that are less than five voxels or lesions appearing on a single slice) or lesions at the border between GM and WM due to imperfect tissue segmentation. However, MSmetrix is quite consistent in segmenting small, medium and large lesions. False positive lesions are mostly found near the frontal horn, in the posterior end of the corpus callosum, also known as splenium, and in the cortical region near the great longitudinal fissure. On dataset 2, the reproducibility of the segmentation as well as the lesion volumes were evaluated, as the reproducibility on the test–retest data is a very important aspect to check the consistency and reliability of the results. The main sources of inconsistency are differences in the estimation of the lesion boundaries between both scans and differences in detecting smaller (often false) lesions (see, e.g., Fig. S4 in the supplementary material).

The comparison with the existing lesion segmentation tools, LST and Lesion-TOADS, shows the outperformance of MSmetrix regarding the accuracy of the lesion segmentation. The Dice similarity index with respect to the expert reference segmentation is significantly higher for MSmetrix than for LST (p < 0.01) and Lesion-TOADS (p < 0.01). Looking in more details, this could mainly be explained by the significantly higher precision compared to LST (p < 0.01) and the significantly higher sensitivity compared to Lesion-TOADS (p < 0.01) as well as LST (p < 0.05). LST, however, has a slightly lower average absolute volume error, although not significantly. This suggests that it misses lesions or hypo-intense lesions (see, e.g., Fig. 4) and compensates for these missed lesions' volume by overestimating other, mostly larger, lesions (see, e.g., Fig. 3) This also explains the increase in segmentation overlap performance of LST as a function of the lesion load. Additionally, LST often introduces false lesions in the corpus callosum (see, e.g., Fig. S1 in the supplementary material). For Lesion-TOADS, the comparable precision but a lower sensitivity compared to MSmetrix, implies a larger amount of false positives within the Lesion-TOADS segmentation. It suffers from false lesion segmentation, which is, unlike MSmetrix, randomly distributed in the image (see, e.g., Fig. S2 in the supplementary material) and is less accurate in segmenting the lesion boundary when compared to MSmetrix. Lesion-TOADS, like LST and MSmetrix, also shows an increase in the segmentation overlap with expert segmentation as a function of lesion load.

The first dataset presented in this paper, including expert reference segmentations, was also used in Steenwijk et al. (2013), which means that it is possible to directly compare results herein to those published in Steenwijk et al. (2013). The results in Steenwijk et al. (2013) may in fact be regarded as a best-case scenario, because the k-nearest neighbour classification employed therein is a supervised classification technique that was trained using a leave-one-out experiment on the 20 patients of dataset 1, based on the available expert reference segmentation. An optimal configuration has been sought and post-processing has been applied to reduce the number of small false positive regions. The average Dice similarity index reached a maximum of 0.75 ± 0.08 after optimal post-processing. Volumetric correspondence in terms of ICC was 0.93 after post-processing. These numbers can be compared to the MSmetrix, LST and Lesion-TOADS results in Table 1, keeping in mind that these methods are unsupervised and did not require any tuning or post-processing on this particular dataset.

Regarding the reproducibility of the lesion segmentation, we found a similar overlap as well as a similar absolute lesion volume difference between MSmetrix and LST. Both automatic methods have almost identical limits of agreement (see, e.g., Fig. 5). In contrast, Lesion-TOADS shows much larger limits of agreement indicating clearly lower lesion volume reproducibility. Similar to MSmetrix, Lesion-TOADS is not very consistent in introducing false positive lesions in two consecutive scans (see, e.g., Fig. 6), and, secondly, it is not as sensitive as MSmetrix in segmenting the lesion boundaries in the test–retest scans (see, e.g., Fig. 6).

In conclusion, the proposed automatic lesion segmentation method, MSmetrix, has been demonstrated to provide more accurate lesion segmentations with a similar reproducibility compared to the state-of-the-art software tools. We believe that, through its robustness and automation, MSmetrix could bring an added value (possibility to measure lesion volumes) for the clinical routine evaluation of MS patients.

## Figures and Tables

**Table 1:** Agreement measures (Dice similarity index, ICC and absolute volume difference) between automatic and expert reference lesion segmentation for MSmetrix, LST and Lesion-TOADS for 20 MS patients.

Automatic method	Dice	ICC	Absolute volume difference (ml)
MSmetrix	0.67 ± 0.11	0.80	5.15 ± 4.75
LST	0.55 ± 0.16**	0.87	4.75 ± 3.63
Lesion-TOADS	0.61 ± 0.09**	0.63	6.86 ± 5.70**

Dice and absolute volume difference are presented in mean ± standard deviation.

** Values significantly different from MSmetrix (paired t-test with p < 0.01 significance level).

**Table 2:** Segmentation quality measures (sensitivity and precision) between automatic and expert reference lesion segmentation for MSmetrix, LST and Lesion-TOADS for 20 MS patients.

Automatic method	Sensitivity	Precision
MSmetrix	0.57 ± 0.13	0.83 ± 0.11
LST	0.50 ± 0.22*	0.70 ± 0.09**
Lesion-TOADS	0.50 ± 0.08**	0.81 ± 0.17

Sensitivity and precision are presented in mean ± standard deviation.

* Values significantly different from MSmetrix (paired t-test with p < 0.05 significance level).

** Values significantly different from MSmetrix (paired t-test with p < 0.01 significance level).

**Table 3:** Agreement measure (average Dice similarity index) for small (n = 3), medium (n = 9) and large (n = 8) lesion volumes for automatic methods. Here, the t-test is not performed, as the sample size is small for each group.

Automatic method	Average Dice		
	(<5) ml	(5–15) ml	(>15) ml
MSmetrix	0.61	0.62	0.74
LST	0.33	0.51	0.69
Lesion-TOADS	0.52	0.58	0.67

**Table 4:** Agreement measures (Dice similarity index and absolute volume difference) between scan 1 and scan 2 of the corresponding automatic methods.

Automatic method	Dice	Absolute volume difference (ml)
MSmetrix	0.69 ± 0.14	0.54 ± 0.58
LST	0.71 ± 0.18	0.44 ± 0.69
Lesion-TOADS	0.63 ± 0.17**	1.58 ± 2.2

Dice and absolute volume difference are presented in mean ± standard deviation.

** Values significantly different from MSmetrix (paired Wilcoxon signed-rank test with p < 0.01 significance level).

## Appendix A Supplementary data

Supplementary material for additional information and supporting figures regarding the accuracy and reproducibility for MSmetrix, LST and Lesion-TOADS.
