# Supplementary material for: Automatic segmentation and volumetry of multiple sclerosis brain lesions from MR images
Source: Neuroimage Clin. 2015 May 16;8:367–75. doi: 10.1016/j.nicl.2015.05.003 (PMC4474324; doi:10.1016/j.nicl.2015.05.003)
Supplement: Supplementary file 1 — Supplementary material for additional information and supporting figures regarding the accuracy and reproducibility for MSmetrix, LST and Lesion-TOADS. [file mmc1.pdf]

7 Supplementary material

Table of contents

BEST CASE FOR LST ON ACCURACY 29

WORST CASE FOR LST AND LESION-TOADS ON ACCURACY 30

BEST CASE FOR LST ON REPRODUCIBILITY 31

BEST CASE FOR LESION-TOADS ON REPRODUCIBILITY 32

WORST CASE FOR LST ON REPRODUCIBILITY 33

WORST CASE FOR LESION-TOADS ON REPRODUCIBILITY 34

This supplementary material provides additional information and supporting figures regarding the accuracy and reproducibility for all methods.

7.1 Best case for LST on accuracy

Figure S.1 shows the best case for LST (Dice: 0.80, sensitivity: 0.80 and precision: 0.79). This case has Dice: 0.82, sensitivity: 0.76, precision: 0.90 for MS**metrix** and Dice: 0.70, sensitivity: 0.54, precision: 0.98 for Lesion-TOADS. The higher sensitivity of LST compared to the other two methods is caused by the detection and segmentation of subtle lesions (marked by a pink arrow

head); however, the lower precision of LST suggests that it introduced false lesions and it overestimated the lesion boundary (see region marked by cyan arrow heads). MS**metrix** has higher Dice similarity index than LST due to lower number of false positive lesion voxels.

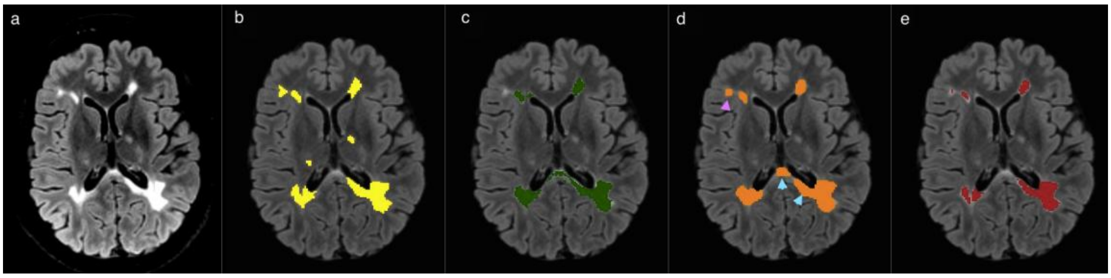

*Figure S.1: Original FLAIR image (a) followed by bias corrected FLAIR image and super-imposed lesion segmentation from: (b) expert segmentation, (c) MS**metrix**, (d) LST, (e) Lesion-TOADS. Cyan arrow heads show false positive lesions and overestimation of the lesion boundaries in LST. Pink arrow head show lesions picked by LST but not by the other methods except partially one in Lesion-TOADS.*

7.2 Worst case for LST and Lesion-TOADS on accuracy

Figure S.2 shows the worst case for LST (Dice: 0.31, sensitivity: 0.21 and precision: 0.58), which is also the worst case for Lesion-TOADS (Dice: 0.44, sensitivity: 0.38 and precision: 0.52). Here, MS**metrix** has a better Dice similarity index, sensitivity and precision compared to the other two methods (Dice: 0.52, sensitivity: 0.40, precision: 0.76). The low sensitivity and precision of LST are due to the fact that it did not find the big lesion, indicated by the purple arrow head in (d). On the other hand, for MS**metrix** and Lesion-TOADS, low sensitivity is due to missing subtle lesions and/or underestimation of lesion boundary (purple arrow head). Lesion-TOADS has a lower precision compared to MS**metrix** because it finds a lot of false positive lesions (cyan arrow head).

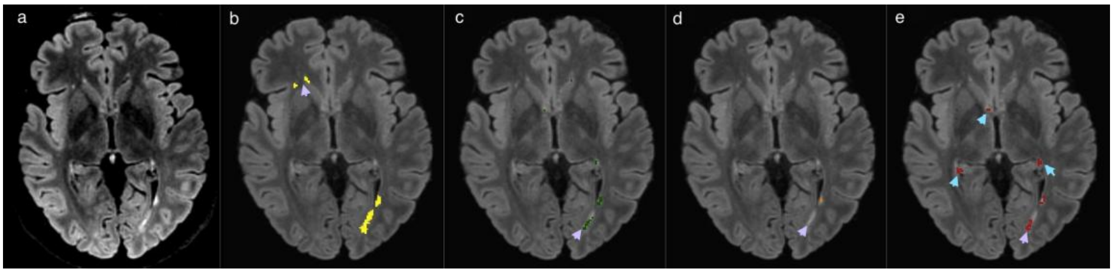

*Figure S.2: Original FLAIR image (a) followed by bias corrected FLAIR image and super-imposed lesion segmentation from: (b) expert segmentation, (c) MS**metrix**, (d) LST, (e) Lesion-TOADS. Cyan arrow heads show false positive lesions in Lesion-TOADS. Purple arrow heads show missed subtle lesions and underestimation of lesion boundary.*

7.3 Best case for LST on reproducibility

Figure S.3 shows the best case for LST (Dice: 0.84). In this case, MS**metrix** has a similar Dice of 0.83 followed by Lesion-TOADS (Dice: 0.78). A lower Dice similarity index for Lesion-TOADS is mainly due to the inconsistent estimation of lesion boundaries (marked by cyan arrow heads) compared to MS**metrix** and LST, which are more consistent in the lesion segmentation in both scans. However, on the other hand, Lesion-TOADS misses big lesions (marked by cyan arrow heads) whereas MS**metrix** and LST detect them successfully.

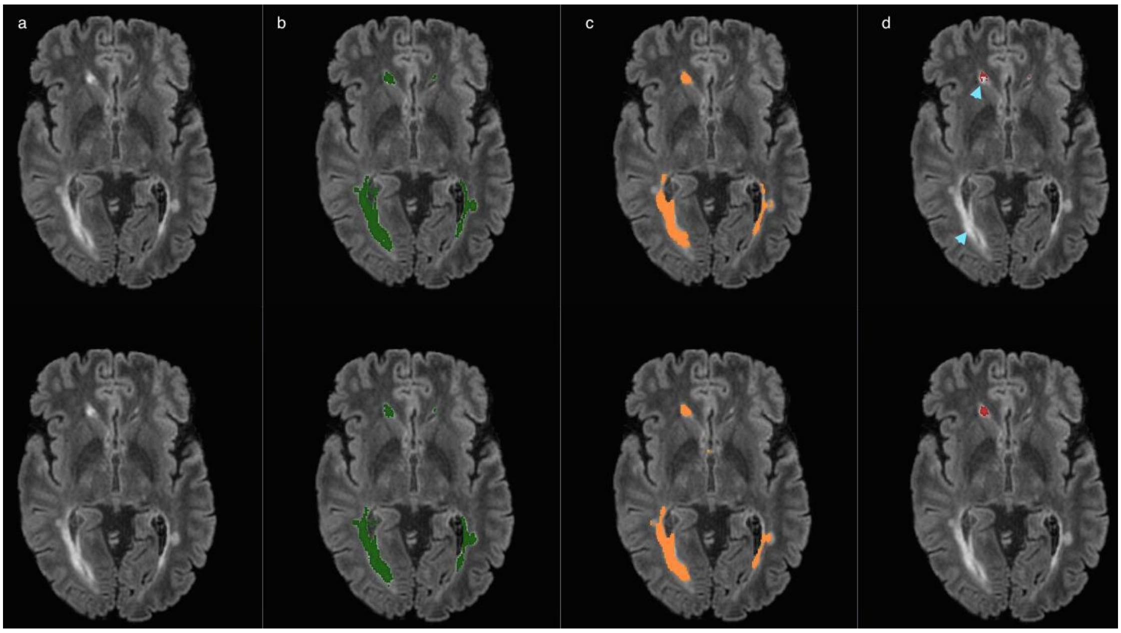

*Figure S.3: Bias corrected FLAIR image (a) and super-imposed lesion segmentation from: (b) MS**metrix**, (c) LST, (d) Lesion-TOADS. The first row corresponds to the lesion segmentation of scan 1 and the second row corresponds to the lesion segmentation of scan 2. Cyan arrow heads show missed lesions and difference in the lesion segmentation boundary between scan 1 and scan 2 for Lesion-TOADS.*

7.4 Best case for Lesion-TOADS on reproducibility

Figure S.4 shows the best case for Lesion-TOADS (Dice: 0.82). In this case, LST and MS**metrix** have comparable Dice of 0.79 and 0.77, respectively. The higher Dice similarity index for Lesion-TOADS compared to MS**metrix** and LST is mainly due to its quite consistent performance in estimation of lesion boundaries in scan 1 and scan 2 for this case. A lower Dice similarity index for both MS**metrix** and LST accounts for (probably) several false lesions in either of the scans (marked by cyan arrow heads).

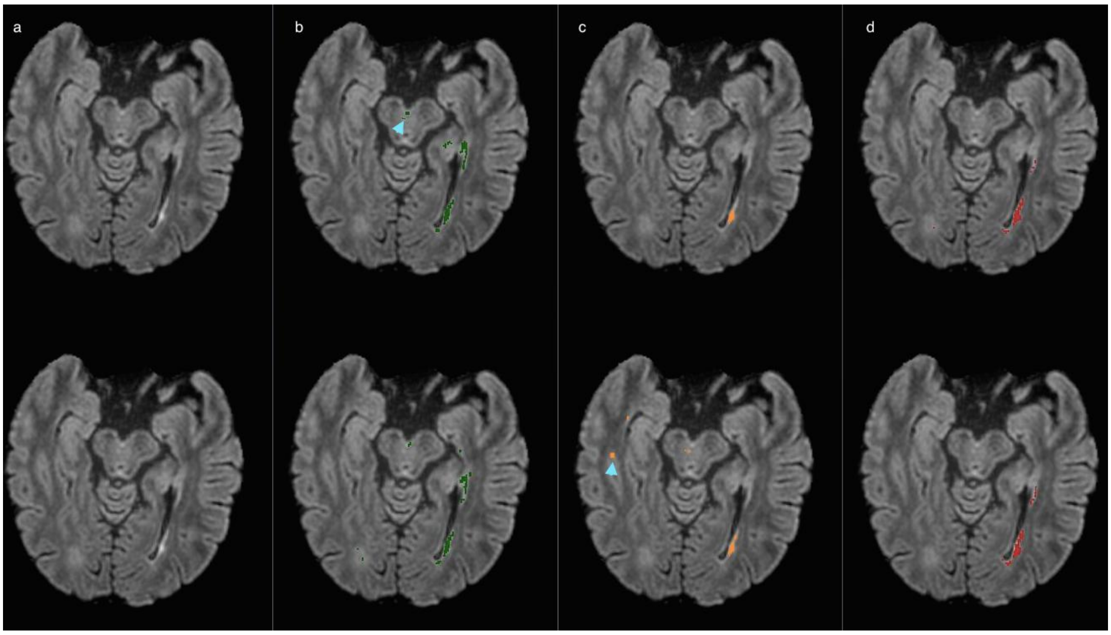

*Figure S.4: Bias corrected FLAIR image (a) and super-imposed lesion segmentation from: (b) MS**metrix**, (c) LST, (d) Lesion-TOADS. The first row corresponds to the lesion segmentation of scan 1 and the second row corresponds to the lesion segmentation of scan 2. Cyan arrow heads represent false lesion detection for MS**metrix** and LST.*

7.5 Worst case for LST on reproducibility

Figure S.5 shows the worst case for LST (Dice: 0). In this case, MS**metrix** and Lesion-TOADS have comparable Dice of 0.38 and 0.35, respectively. Before we explain the results, its important to mention here that this subject has very few lesions. A zero Dice similarity index for LST is primarily due to the fact that it is unable to find any lesions in scan 1, but it finds some lesions in scan 2. Both MS**metrix** and Lesion-TOADS consistently find both true lesions (pink arrow head) and false lesions (cyan arrow head) across the scans. However, the lower Dice similarity index for Lesion-TOADS accounts for slightly more false lesion detection in either of the scans compared to MS**metrix**.

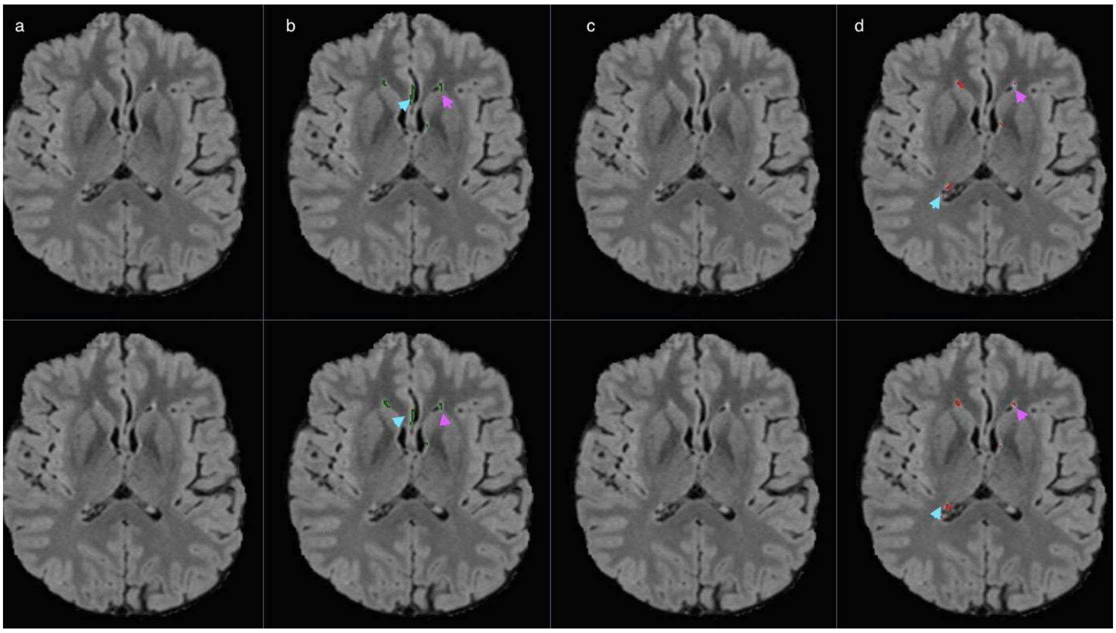

*Figure S.5: Bias corrected FLAIR image (a) and super-imposed lesion segmentation from: (b) MS**metrix**, (c) LST, (d) Lesion-TOADS. The first row corresponds to the lesion segmentation of scan 1 and the second row corresponds to the lesion segmentation of scan 2. Cyan arrow heads show false lesions detection for MS**metrix** and Lesion-TOADS. Pink arrow heads show subtle lesions that are picked up by MS**metrix** and Lesion-TOADS.*

7.6 Worst case for Lesion-TOADS on reproducibility

Figure S.6 shows the worst case for Lesion-TOADS (Dice: 0.15). In this case, LST has the best performance (Dice: 0.63) followed by MS**metrix** (Dice: 0.40). The lower Dice similarity index for Lesion-TOADS is primarily due to the fact that quite some non-overlapping lesions are found, which are probably false positives (cyan ellipse). For LST, the higher Dice similarity index is mainly because it consistently finds lesions across the scans. A low Dice similarity index for MS**metrix** is mainly due to some false lesion detection in either of the scans (marked by cyan arrow heads). Although the Dice similarity index is higher for LST compared to MS**metrix**, LST is slightly imprecise in lesion boundary estimation (marked by cyan arrow heads) for this case.

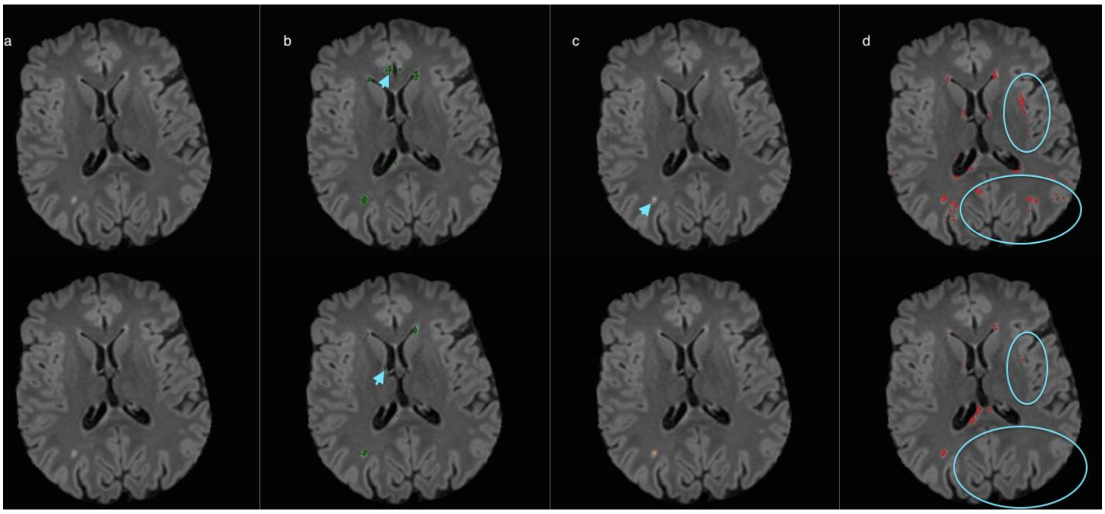

*Figure S.6: Bias corrected FLAIR image (a) and super-imposed lesion segmentation from: (b) MS**metrix**, (c) LST, (d) Lesion-TOADS. The first row corresponds to the lesion segmentation of scan 1 and the second row corresponds to the lesion segmentation of scan 2. Cyan ellipses represent the non-overlapping lesions between scan 1 and scan 2 for Lesion-TOADS, which are probably false positives. Cyan arrow heads show false lesion detection by MS**metrix** and imprecise lesion boundary estimation by LST in both scans.*
